# Controlled Delivery of 2-Mercapto 1-Methyl Imidazole by Metal–Organic Framework for Efficient Inhibition of Copper Corrosion in NaCl Solution

**DOI:** 10.3390/ma16206712

**Published:** 2023-10-16

**Authors:** Zhishun Zhu, Xiulan Zhang

**Affiliations:** Key Laboratory for Green Chemical Process of Ministry of Education, School of Chemical Engineering and Pharmacy, Wuhan Institute of Technology, No. 206, Guanggu 1st Road, Donghu New & High Technology Development Zone, Wuhan 430205, China

**Keywords:** corrosion inhibitor, ZIF-8, loading and delivery, AMOF, theoretical calculation

## Abstract

In this paper, zeolitic imidazolate framework-8 was modified by N-(3-aminopropyl)-imidazole to obtain a novel MOF called AMOF. Subsequently, AMOF served as a carrier for the delivery of 2-mercapto-1-methyl imidazole (MMI) to inhibit the corrosion of Cu. Scanning electron microscopy, Fourier transform infrared spectroscopy, and X-ray diffraction were applied to characterize the morphologies and structures of AMOF and AMOF@MMI. Ultraviolet-visible spectroscopy and thermogravimetric analysis were adopted to value the capacity of the load and release of the AMOF, respectively. The mass ratio of loaded MMI molecules was 18.15%. In addition, the inhibition behavior of AMOF@MMI for Cu was evaluated by polarization curves and electrochemical impedance spectroscopy. The results indicated that the AMOF loaded MMI successfully, and the released MMI could adsorb on the Cu surface and inhibit the Cu corrosion. The inhibition efficiency could reach 88.2%. The binding and interaction energies between the AMOF surface and the MMI were −16.41 kJ/mol and −20.27 kJ/mol.

## 1. Introduction

Cu is a widely used industrial metal because of its excellent thermoelectrical conductivity, mechanical workability, and malleability. However, the corrosion of Cu hampers its wide range of applications, such as in the oil industry, marine equipment, electronic materials, and transportation [1,2,3,4,5]. More severely, the corrosion of Cu metal not only affects the performance of the metal and causes huge economic losses but also poses inestimable safety risks [6]. The utilization of corrosion inhibitors including imidazole [7], quinolones [8], mercaptans [9], thiazides [10], and amino acids [11], which generally contain nitrogen, oxygen, sulfur, and heterocycles [7,12,13] and are an efficient strategy for protecting Cu from corrosion. Among these inhibitors, imidazoline derivatives have become the most applied type, especially in oil and gas facilities, because of their excellent anti-corrosion performance and low toxicity [14]. However, the continuous and increasing consumption of corrosion inhibitors in aquatic environments not only wastes extra inhibitors but also pollutes the environment. Researchers have attempted to overcome this problem by using a controlled release system [15,16,17,18], and many inhibitor containers have been designed and prepared in recent years [19]. Halloysite nanotubes, layer double hydroxides, metal–organic frameworks (MOFs), and clay particles are well-known containers because of their extended porosity and large specific surface area properties [20,21,22]. Among the various kinds of containers, MOFs, which are composed of metal ions connected by organic linkers, have received considerable attention as drug-delivery nanocarriers because of their important loading capability, biocompatibility, and convenient synthesis under mild conditions [23,24,25,26]. As a typical class of MOFs, zeolitic imidazolate framework-8 (ZIF-8) possesses a remarkable opportunity for drug release due to its additional vacancy coordination, exposed defects, and functional groups [27,28,29]. The application of MOFs as a container for corrosion inhibitor loading has also been investigated. Yang et al. [30] investigated an anti-corrosion system through a one-pot method by wrapping benzotriazole into ZIF-8. Their investigation revealed that ZIF-8 had a high drug loading rate and that the ZIF-8/benzotriazole system had superior anti-corrosion performance and could effectively enhance the protective performance of the coating. Tian et al. [31] synthesized a triazole-based inhibitor, which controlled delivery by ZIF-8, to inhibit mild steel corrosion in a NaCl solution. Xiao et al. [32] successfully synthesized Ce-IM/ZIF-8 NPs, which showed apparent pH stimuli-responsive release ability, could release Ce^3+^, Zn^2+^ ions and imidazole upon acid stimulus, and a Ce^3+^ ions and imidazole presented synergistic inhibition effect on Al corrosion.

In recent years, density functional theory (DFT) has been widely applied to reveal the interaction mechanism at the molecular level [33,34,35,36,37]. The molecular structures, binding sites, and binding energies have been revealed by DFT calculations for the adsorption of metals and organic chemicals onto various materials [32,35,38,39,40]. Sun et al. [35] investigated the adsorption mechanisms of ibuprofen and naproxen among MOFs through DFT calculations. The binding energies followed the order of π-π > hydrogen bonding > LAB > anion-π. Javidparvar et al. [34] examined the interactions and adhesion of Ce^3+^ over the graphene oxide surface by using electronic ab initio quantum mechanics approaches at detailed electronic scales. Their results demonstrated the physisorption and chemisorption of Ce^3+^ onto the graphene oxide surface. 

In this paper, we first synthesized AMOF. Then, AMOF@MMI was obtained by stirring the MOF particles in ethanol solution that contained the MMI inhibitor. The prepared AMOF@MMI was characterized with scanning electron microscopy (SEM), energy dispersive spectrometry (EDS), X-ray diffraction (XRD), Fourier transform infrared spectroscopy (FTIR), and N_2_ adsorption–desorption isotherms. The inhibitor uptake and release were followed by UV-vis adsorption spectra and TGA. The potential adsorption mechanisms between the AMOF and MMI were studied in detail by DFT calculations. The inhibition performance was evaluated by electrochemical impedance spectroscopy (EIS) and potentiodynamic polarization curves. Through this work, the loading and release mechanisms of AMOFs on the corrosion inhibitor MMI will be revealed.

## 2. Experimental

### 2.1. Materials and Solutions

Zn(NO_3_)_2_·6H_2_O (99%) and N-(3-aminopropyl)-imidazole (99%) were purchased from the Shanghai Sigma–Aldrich Company Ltd. (Shanghai, China). MMI was purchased from the Shanghai Aladdin Company Ltd. (Shanghai, China). All reagents were used as received without further modification.

Cu electrodes with an exposed area of 1 cm^2^ were polished with #800 to #2000 sandpapers, and cleaned in acetone and ethanol under ultrasonic conditions. The chemical composition (wt.%) of the copper used for the research was as follows: 0.005% Zn, 0.003% Pb, 0.001% B, 0.002% As, 0.002% Sb, 0.005% Ni, and Cu (remaining proportion).

### 2.2. Preparation of AMOF and AMOF@MMI 

AMOF was prepared according to a previously reported method [30]. A total of 1.249 g Zn(NO_3_)_2_·6H_2_O was dissolved in 35 mL of absolute methanol solution. Meanwhile, 2.0 mL N-(3-aminopropyl)-imidazole was also dissolved in 35 mL of absolute methanol solution. Subsequently, the two solutions were mixed in a beaker and stirred vigorously for 30 min. The white turbid solution was sealed into a Teflon-lined autoclave and maintained in a pre-heated oven at 140 °C for 24 h. After cooling to room temperature, the white powder product was obtained by ethanol washing and vacuum drying at 60 °C for 12 h. 

The obtained AMOF nanoparticles (1.0 g) were dispersed in a solution that contained 200 mL ethanol and 3.0 g MMI. Next, the solution was sonicated for 10 min. Then, the solution was transferred to a round-bottomed flask and stirred vigorously for 24 h at 25 °C under vacuum. Subsequently, the precipitate was centrifuged at 5000 rpm, washed with ethanol several times, and dried at 60 °C in a vacuum oven overnight. The obtained nanoparticles were denoted as AMOF@MMI. 

### 2.3. Characterization of AMOF and AMOF@MMI

The surface morphologies of AMOF and AMOF@MMI were observed by SEM (SU8010HHTNT-536-9424SU8010, Hitachi Limited, Fukuoka-shi, Japan). The ATR-FTIR spectra of the samples were recorded in the range of 4000–400 cm^−1^ by a Fourier transform infrared spectrometer (VERTEX808000-350 cm^−1^ VETEX 80, Bruker, Bremen, Germany). The crystal structures of the samples were characterized by XRD patterns with Cu-*K* alpha in the 2θ range of 5°–60° at a scan step of 5°/min (Smart Lab-SE, JESCO, Yokohama, Japan). The loading capacity of the AMOF was evaluated by a TGA (Diamond TG/DTA, PerkinElmer Instruments, Shelton, CT, USA) between 25 °C and 800 °C under an air atmosphere (100 mL/min), and the heating rate was 10 °C/min. The MMI concentration in the supernatant after centrifugation and its release amount was determined by a UV-vis spectrophotometer (UV-2550 spectrometer, Shimadzu, Kyoto, Japan). A total of 100 mg of AMOF@MMI was immersed in 1000 mL of 0.5 M NaCl solution for slow release under continuous stirring. At different times, one supernatant was recovered by filtration using a 0.22 μm micro porous cellulose membrane and replaced with the same volume of fresh culture medium [31]. 

Kinetic models, such as pseudofirst-order models, were used to investigate the release mechanism of the MMI. Equation (1) describes the pseudofirst-order as follows [41]:

(1) $$Q_{t}=Ae^{-kt}+Q_{e},$$

where $Q_{e}$ (mg/L) and $Q_{t}$ (mg/L) are the equilibrium and time-dependent amounts of the MMI release at time *t* (h), respectively. *A* is a pre-exponential factor. *k* (h^−1^) is a constant of a pseudo-first-model rate. 

The surface areas of the samples were obtained on a fully automatic surface area and porosity analyzer (ASAP2420-4MP*, Mac America, Paso Robles, CA, USA). Prior to the analysis, the bared AMOF and AMOF@MMI were dehydrated and evacuated for 12 h at 70 °C under vacuum. The Brunauer–Emmett–Teller (BET) surface was estimated at a relative pressure lower than 0.25. XPS (AXIS-ULTRA DLD-600 W XPS, Shimadzu-Kratos, Kyoto, Japan) was used to determine the presence of the oxide and organic layers and the elemental composition in the first few nanometers of the Cu surface.

### 2.4. Electrochemical Measurements

Electrochemical measurements were carried out using an electrochemical workstation (Interface 1010B, Gamry, Warminster, PA, USA) at 25 °C in a conventional three-electrode cell, and the working electrode was Cu. A platinum sheet and a saturated calomel electrode (SCE) were used as the counter and reference electrodes, respectively. All potentials were quoted to the SCE. After the stabilization of the open circuit potential (OCP) (OCP changing less than 2 mV/min), potentiodynamic polarization measurements were carried out from −0.2 V to +0.2 V vs. OCP at a sweep rate of 0.5 mV/s. The inhibition efficiency (η) was calculated using the corrosion current density data given by Equation (2):

(2) $$\eta =\frac{i_{corr}^{0}-i_{corr}}{i_{corr}^{0}}\times 100,$$

where $i_{corr}^{0}$ and $i_{corr}$ are the corrosion current densities for the Cu electrode in the 0.5 M NaCl solution without and with the MMI inhibitor, respectively. EIS measurements were performed at different immersion times. The system was perturbed by sinusoidal waves at 5 mV amplitude at frequencies that varied from 100 kHz to 10 mHz, 5 points per decade. To ensure the reproducibility of the test results, each experiment was conducted three times and the average value was taken.

### 2.5. Quantum Chemical Calculations 

DFT is an economical and efficient quantum chemical calculation method that provides sufficiently accurate information, such as geometry and electron distribution [42]. It has been widely used in inhibitor performance analysis and inhibitor/interface interaction. In this paper, quantum chemical calculations were performed using Gaussian 09 W software employing DFT. The MMI molecule was fully optimized using the B3LYP method at the DFT level with a 6–31^+^ G (d) basis set. Subsequently, some parameters, including the energy of the highest occupied molecular orbital (E_HOMO_), energy of the lowest unoccupied molecular orbital (E_LUMO_), energy gap (ΔE = E_LUMO_ − E_HOMO_), and dipole moment (μ) were calculated. It could also compute the interaction between the AMOF structure and the MMI [34].

In this paper, molecular dynamics (MD) simulation was carried out by using the Forcite mode in a Material Studio 8.0 software package. The bulk structure of Cu was fully optimized, and a 6 × 6 supercell of Cu (111) surface that comprised two layers with 72 Cu-atoms (slab thickness of 3 Å) was created. The Cu surface was created by introducing a 30 Å vacuum along the z-direction. MD simulation was performed at 298 K maintained constant by the Andersen thermostat, a time step of 1 fs, canonical ensemble, and a simulation time of 5000 ps to reach the simulation system under an equilibrium state. The extent of the interaction of the inhibitor molecule adsorbed on the Cu surface was demonstrated by their interaction (E_interaction_) and binding (E_binding_) energies derived using Equations (3) and (4), respectively:

E_interaction_ = E_total_ − (E_surface+solution_ + E_inhibitor_), (3)

E_interaction_ = –E_binding_, (4)

where E_total_, E_surface+solution_, and E_inhibitor_ are the total energies of the optimized AMOF@MMI, Cu the surface and solution, and the isolated molecule, respectively [7].

## 3. Results

### 3.1. Characterization of AMOF and AMOF@MMI

**Figure 1:**
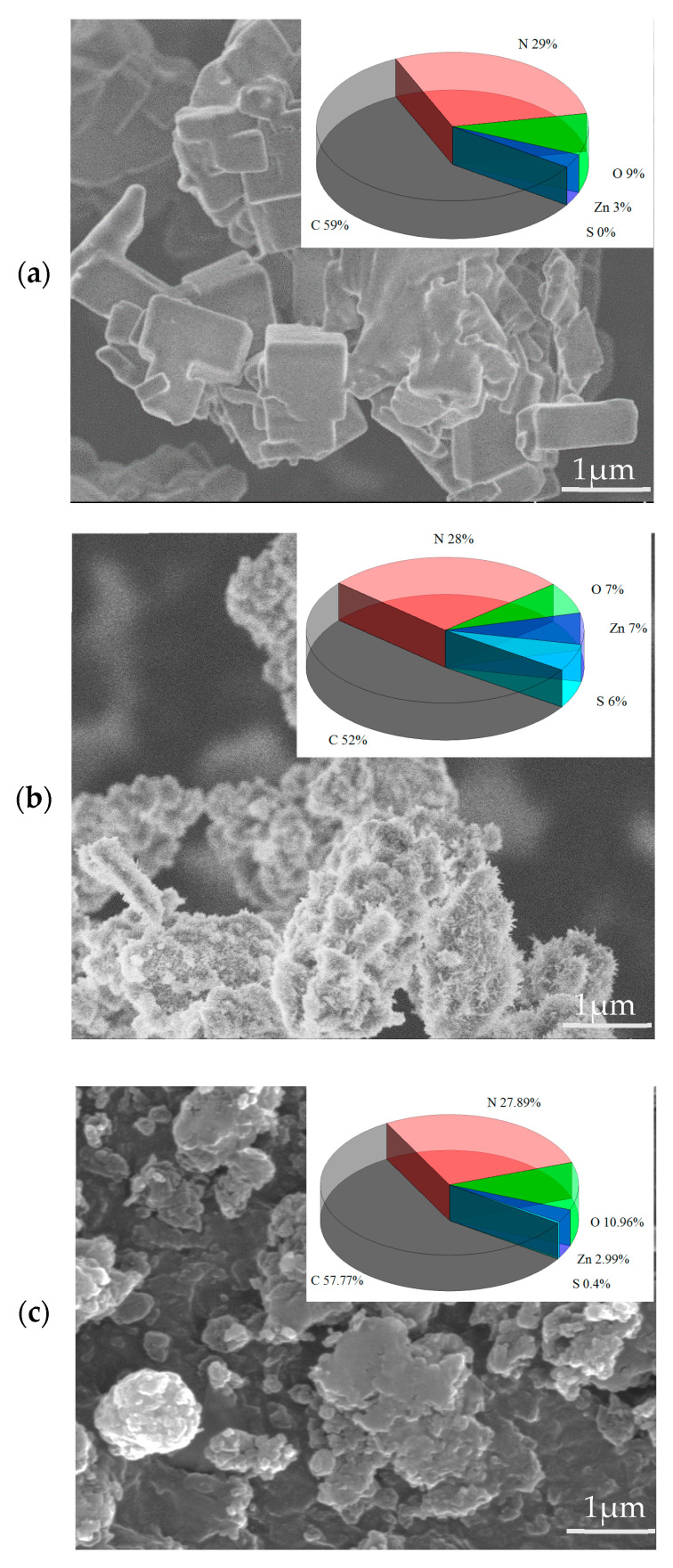
SEM images of (**a**) AMOF, (**b**) AMOF@MMI, (**c**) AMOF@MMI after MMI release.

The surface morphologies of the AMOF and AMOF@MMI are presented in Figure 1. The SEM image of the AMOF showed its regular, smooth, and well-defined cuboid morphology and size (Figure 1a). It observed a cuboid structure covered with small fine needles for the AMOF@MMI (Figure 1b). Figure 1c illustrates that the AMOF itself had a smooth surface after the release of the MMI. The EDS results (Figure 1b inset) showed that the sulfur element of the MMI was evenly distributed on the AMOF surface, demonstrating that the AMOF loads the MMI successfully. Furthermore, at the end of the release process, a very low amount of sulfur was detected in the samples, indicating that the MMI was almost completely released [16]. 

**Figure 2:**
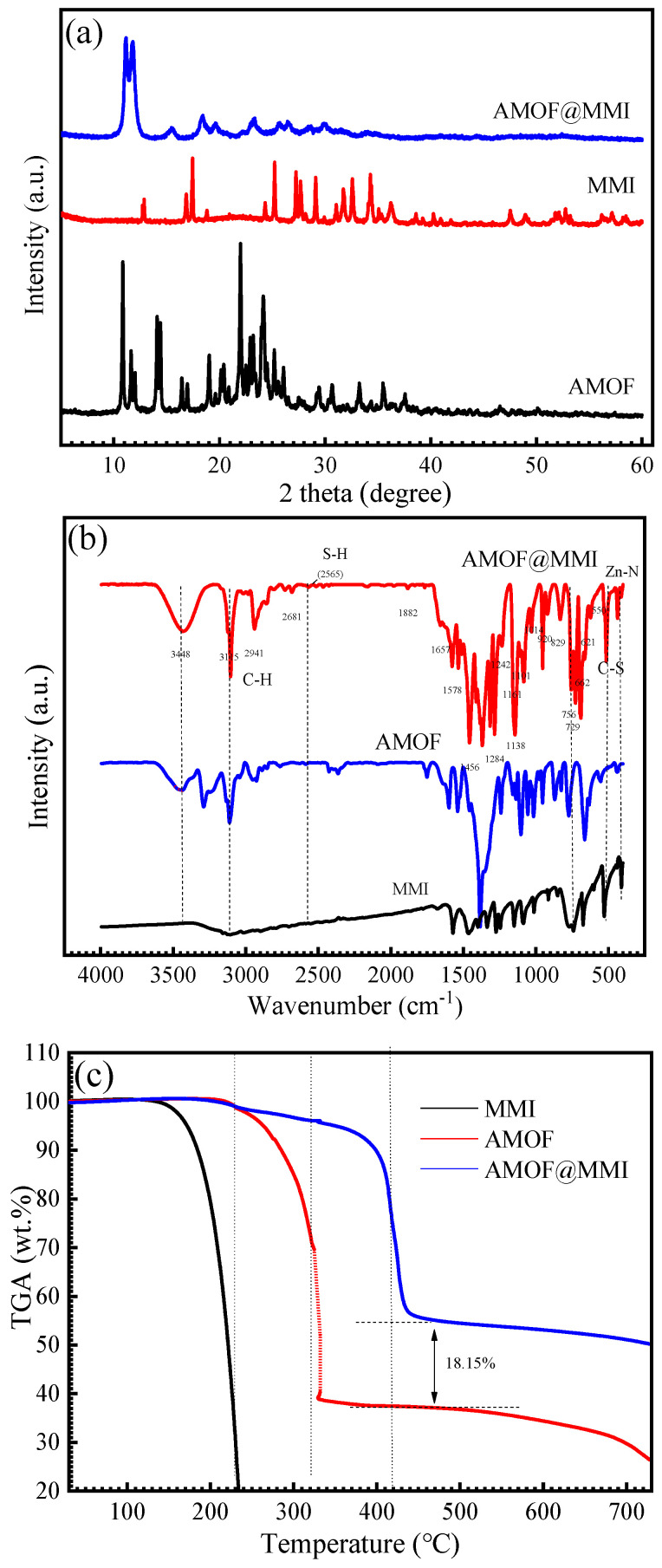
Characterization of AMOF and AMOF@MMI (**a**) XRD spectra, (**b**) FTIR spectrum, (**c**) TGA.

The XRD pattern (Figure 2a) exhibited that AMOF had a crystalline structure, and the XRD pattern of the AMOF@MMI was almost unchanged compared with that of AMOF when loaded with the MMI, indicating that the highly ordered crystalline structures are maintained [31]. The chemical bonds of AMOF and AMOF@MMI were characterized by FTIR (Figure 2b). The characteristic bands in the range of 1350–900 cm^−1^ were assigned to various vibration modes in the imidazole ring. Subsequently, the signal peak at 421 cm^−1^ was attributed to the stretching mode of the Zn–N bond. These findings endorse the theory that the precision of AMOF has been synthesized [43,44]. The spectrum of the MMI sample displayed two strong bands at 3125 cm^−1^ and 2941 cm^−1^ that corresponded to C−H bond vibrations and a band at 2565 cm^−1^ that corresponded to S−H bond stretching vibrations [45]. The characteristic bands in the range of 1625–1400 cm^−1^ were assigned to C−C and C=C bonds’ stretching vibrations. Furthermore, the peaks at 1451 cm^−1^ were assigned to C−N stretching vibrations [45,46]. Therefore, the characteristic bands that appeared in AMOF@MMI confirmed that the AMOF loads the MMI successfully.

The TGA results of the MMI, AMOF, and AMOF@MMI samples are reflected in Figure 2c. For the MMI sample, one significant weight loss only occurred in the 160–240 °C temperature region. This finding was in accordance with the fact that the weak chemical bonds in the imidazole ring decomposed into their previous state. However, the pyrolysis rate in the AMOF decreased exceptionally due to the strong interaction between the Zn (II) and the organic group. TGA results proves that the strong interaction between the AMOF and MMI results in a highly thermally stable AMOF@MMI [47], and the loading capacity of the AMOF achieved 18.15 wt.%. 

**Figure 3:**
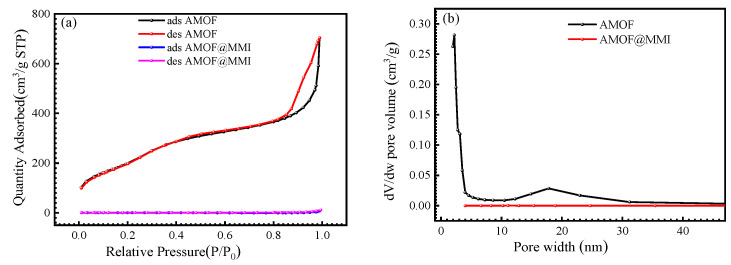
(**a**) N_2_ adsorption/desorption isotherms of AMOF and AMOF@MMI, (**b**) the pore size distribution of AMOF and AMOF@MMI.

Figure 3a depicts the N_2_ adsorption and desorption isotherm curves of the AMOF and AMOF@MMI. The AMOF belonged to the type III absorption according to the IUPAC classification [44,48,49]. For the AMOF microsphere, a certain number of micro pores was distributed in the low relative pressure region, whereas mesoporous structures existed in the medium pressure region. However, when the AMOF loaded MMI molecules, a slight adsorption phenomenon appeared at low or medium relative pressure, indicating that the micropores and mesopores of the AMOF were filled with MMI molecules. Additionally, the special BET surface area was calculated through the N_2_ adsorption–desorption isotherm. After the loading of the MMI, the specific surface area reduced from 713.2 m^2^/g to 2.9 m^2^/g. The pore volume decreased from 0.025 to approximately 0 cm^3^/g with a pore diameter that ranged from 10 to 35 nm (Figure 3b), thereby proving that the MMI approximately filled up the pores and channels, and that this MOF has a remarkable loading capacity of an MMI inhibitor.

### 3.2. Release of AMOF@MMI

**Figure 4:**
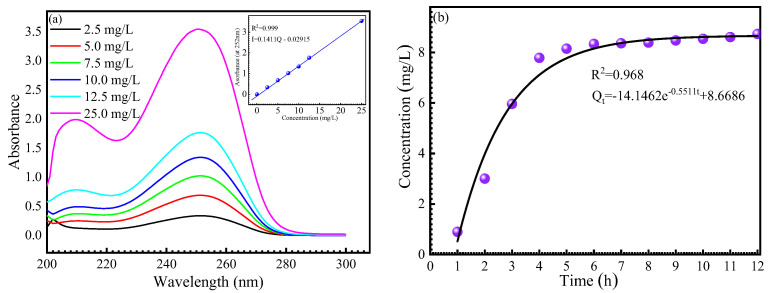
UV-vis absorption spectra of AMOF@MMI (**a**) standard curve, (**b**) kinetics model for release of MMI.

The UV-vis analysis enabled us to understand the release of the MMI from the AMOF container. Figure 4a shows the standard curves of the MMI molecule obtained by the absorbance values at 252 nm. Figure 4b displays the kinetic release profiles of the MMI molecule in 0.5 M NaCl solution. A progressive and sustained release with no burst effect, which consisted of two distinct stages with successively declining release rates, was observed. Next, the remaining fraction was slowly achieved, as illustrated by the following plateau in the release kinetics. The kinetics of the MMI inhibitor delivery were empirically adjusted using the regression factors of nearly 0.97 to a first-order model. In this case, the release of MMI molecules was usually very fast in the first 4 h and then slower until the equilibrium was reached because of the large surface area of and numerous pores on the AMOF surface. 

### 3.3. Potentiodynamic Polarization

**Figure 5:**
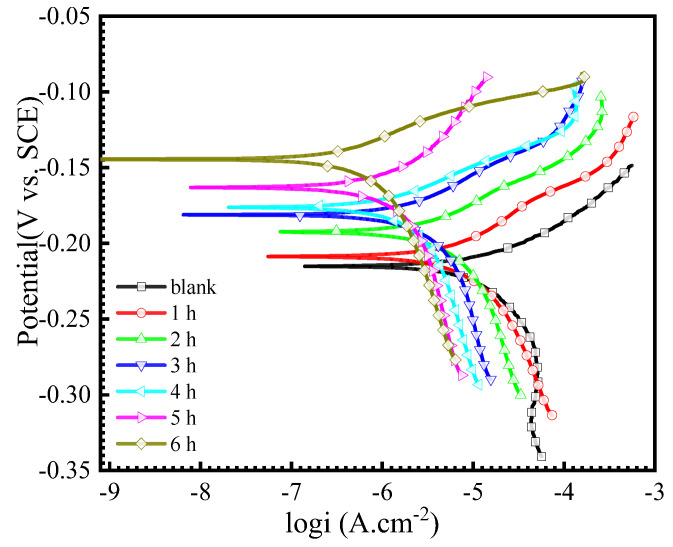
Polarization curves of copper in 0.5 M NaCl with AMOF@MMI at different time at 25 °C.

Figure 5 illustrates the polarization curves for Cu in 0.5 M NaCl solution with 50 mg/L AMOF@MMI at different times. The obtained parameters, such as the corrosion potential (E_corr_), corrosion current density (i_corr_), anode Tafel slope (ba), cathode Tafel (bc), and inhibition efficiency (η), were listed in Table 1. As shown in Figure 5 and Table 1, i_corr_ of the Cu decreased gradually after the immersion in 0.5 M NaCl solution that contained AMOF@MMI for 1, 2, 3, 4, 5, and 6 h. Accordingly, the inhibition efficiency increased from 55.2% (1 h) to 88.2% (6 h). Therefore, the AMOF@MMI system has slow-release capability and the released MMI exhibited good corrosion resistance.

### 3.4. EIS Measurements

**Figure 6:**
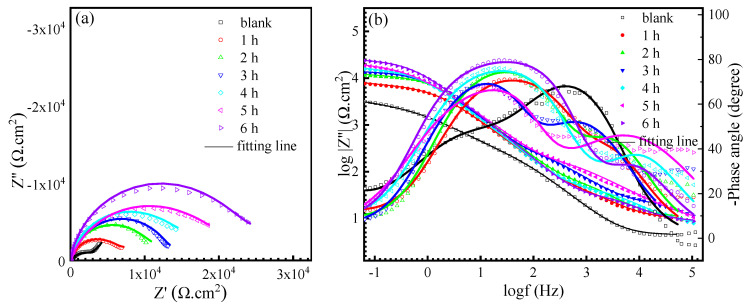
EIS for copper in 0.5 M NaCl with AMOF@MMI (**a**) Nyquist plot, (**b**) Bode plot.

**Figure 7:**
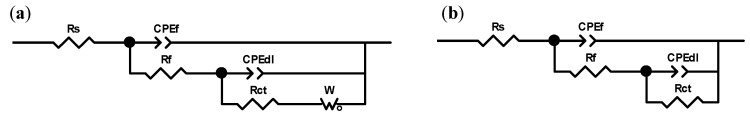
Equivalent circuit diagram of impedance spectra (**a**) blank, (**b**) MMI.

The EIS of Cu was measured to further verify the inhibition effect of AMOF@MMI. Figure 6a,b presents the Nyquist and Bode plots of Cu recorded in a blank solution and after 1, 2, 3, 4, 5, and 6 h of immersion in the NaCl solution with AMOF@MMI, respectively. The equivalent circuits utilized to fit the EIS are shown in Figure 7a,b and the fitting parameters are listed in Table 2. The circuit elements including the Rs, Rct, RL and Rf are assigned to the solution resistance, charge transfer resistance, inductive resistance and film resistance, respectively. Given the imperfect capacitive semicircle, which is due to the dispersion effect with incongruous homogeneousness and roughness of the electrode surface, the ideal capacitance is replaced using the constant phase element (CPE), while the CPEdl and CPEf arise from the double-layer capacitance and film capacitance separately, and L is the Warburg impedance. Figure 6a displays that the Nyquist plots of Cu immersed in the NaCl solution with AMOF@MMI have a larger semicircular diameter than that of the blank one, thereby denoting the evident corrosion inhibition performance of AMOF@MMI. Table 2 shows that R_f_ and R_ct_ values increased and CPE_dl_ decreased over time, suggesting the gradual release of the MMI inhibitor from the AMOF. 

### 3.5. SEM and EDS Analyses

**Figure 8:**
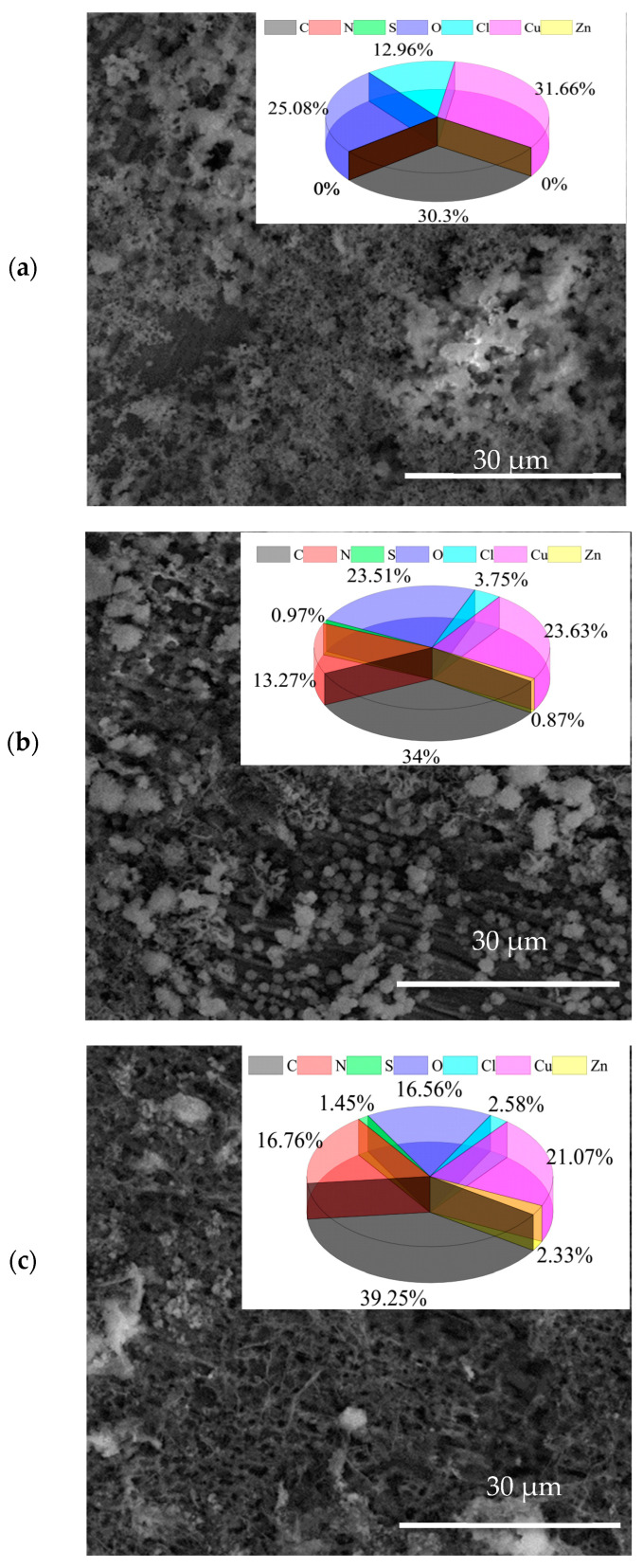
SEM images of copper substrate immerse in (**a**) corrosive 0.5 M NaCl solution, (**b**) AMOF@MMI after 3 days, (**c**) AMOF@MMI after 6 days.

SEM tests are conducted after immersion in 0.5 M of NaCl solution without an inhibitor for 3 h and in the solution with 100 mg/L of AMOF@MMI for 3 h and 6 h. Figure 8a shows that the Cu was severely damaged and covered with loose corrosion products as it was immersed in the blank solution. Conversely, smooth surfaces were clearly observed in Figure 8b,c after the addition of AMOF@MMI. Meanwhile, the EDS results showed that the contents of sulfur and nitrogen gradually increased with the extension of the release time, indicating that the strong adsorption of MMI on Cu surface can effectively inhibit the corrosion of Cu.

### 3.6. FTIR Analysis

**Figure 9:**
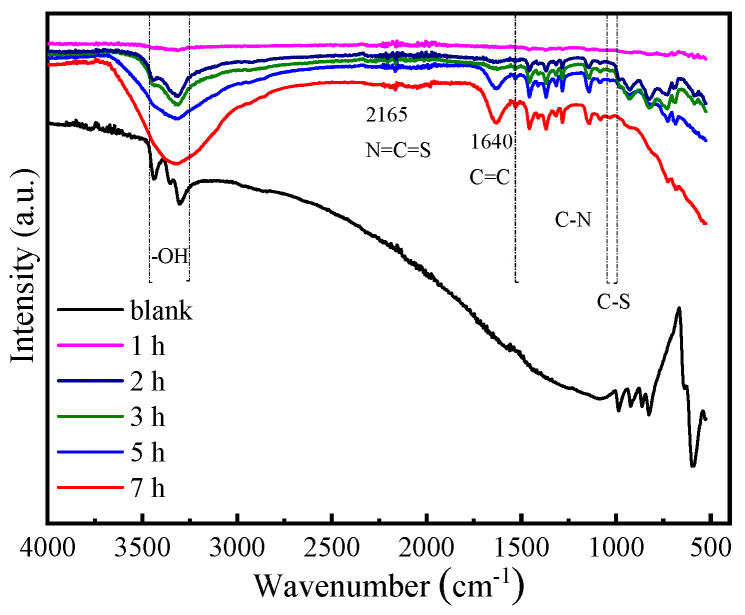
FTIR spectra of copper samples immersed in 0.5 M NaCl solution with or without the addition of AMOF@MMI at different times.

FTIR spectra explored the interaction between the Cu and MMI (Figure 9). Compared with the blank solution, in the presence of AMOF@MMI, the peaks in the range of 2500–2000 cm^−1^ were assigned to the in-plane N=C=S bending of the MMI ring [50]. The absorption peak of 1640 cm^−1^ was assigned to the C=C stretching vibration of the imidazole ring [51]. The peaks at 1457, 1405, 1357, and 1136 cm^−1^ were attributed to the C–N bonding of the corrosion inhibitor [45,46]. The peaks at 1020 cm^−1^ were assigned to C–S stretching [52]. Based on this observation, these spectra clearly show that MMI is adsorbed on the Cu surface. 

### 3.7. XPS Analysis

**Figure 10:**
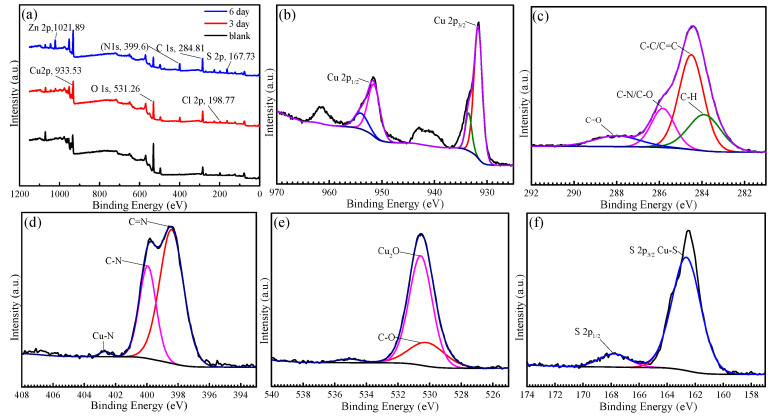
(**a**) XPS survey spectrum of the as-synthesized AMOF@MMI and the related high-resolution spectra of (**b**) Cu 2p, (**c**) C 1s, (**d**) N 1s, (**e**) O 1s, (**f**) S 2p.

XPS analysis was performed on the Cu sample after the immersion in 0.5 M NaCl solution without inhibitor for 3 h and in the solution with 100 mg/L AMOF@MMI for 3 h and 6 h. The chemical composition of the Cu surface, as deduced from the XPS spectra (Figure 10a), is summarized in Table 3. The blank sample, which is compatible with the formation of an oxide layer, contained 32.08 at. % Cu and 28.6 at. % O. The presence of 20.06 at. % C and 7.42 at. % Cl was attributed to an adventitious carbon and chloride contamination. Upon their exposure to the solution with 100 mg/L of AMOF@MMI, the concentration of Cu and O at the surface diminishes together with the trend of increased concentration of C, N, and Zn with time.

High-resolution Cu 2p spectra are exhibited in Figure 10b. In general, the Cu 2p spectrum of pure Cu was composed of two peaks (i.e., a Cu 2p_3/2_ peak at 951.52 e V and a Cu 2p_1/2_ peak at 931.70 eV) [53]. The Cu 2p spectrum determined Cu (II) species present on a Cu surface [54]. C 1s spectra are exhibited in Figure 10c. The peaks at 283.82, 284.38, 285.83, and 287.96 eV were due to C–N, C=C, C=N, and C–O, respectively [50,53]. N 1s spectra are exhibited in Figure 10d. The peaks at 399.94 eV and 398.41 eV were assigned to C–N and N–H bonds, respectively [55]. Moreover, the peak at 402.67 eV represented a Cu–N bond, and its appearance indicates the coordination between the metal and nitrogen contained in the heterocyclic ring [50]. O 1s spectra are exhibited in Figure 10e. The peaks at 530.21, 530.33, and 534.99 eV implied the appearance of Cu_2_O and C–O [56]. S 2p spectra are exhibited in Figure 10f. A single peak was observed at 162.63 eV, which corresponded to a C–S or a C=S group. The results were consistent with the reported values of the mercapto complexes with metals [57]. The peak at 167.79 eV represented an O–S bond, indicating that the thiol group was oxidized for the MMI adsorbed on the Cu surface [55]. 

### 3.8. Theoretical Calculation Results

**Figure 11:**
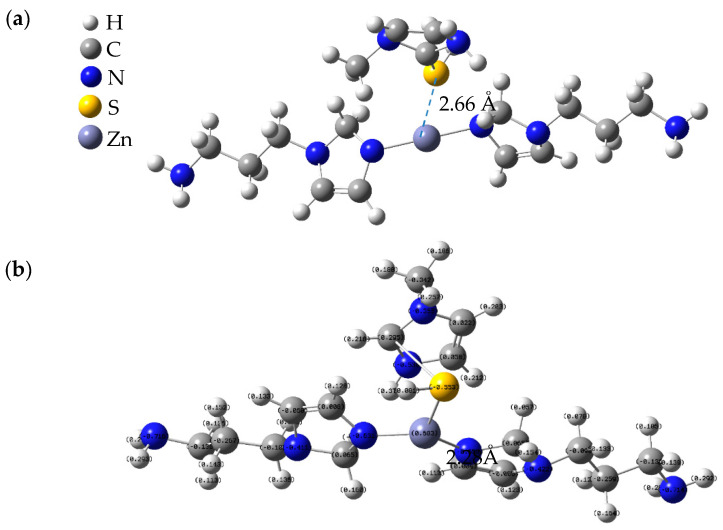
The interaction of MMI with AMOF (**a**) initial geometry of AMOF@MMI, (**b**) final optimized geometry of AMOF@MMI.

The interaction of MMI and AMOF was assessed using DFT tools. Figure 11 reports the designed clusters and the final computed ones extracted from the DFT. The MMI was finally closer to the AMOF surface because of the electrostatic attraction of negatively charged S atoms with positively charged zinc (II) cation located in its surface. The affinity of metal−organic coordination species was investigated further with the prediction of the E_binding_ parameter. The binding energy was −16.41 kJ/mol. The values of the calculated binding energy further prove the MMI adsorption on AMOF surface quantitatively [32,40].

**Figure 12:**
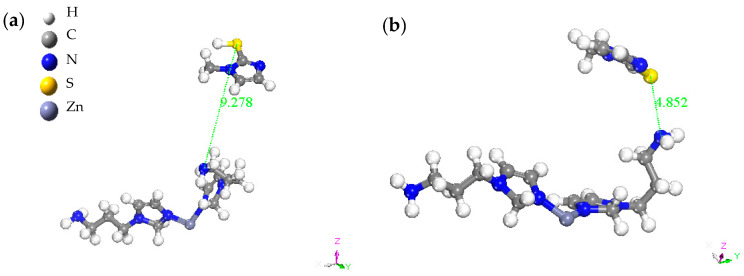
Equilibrium configurations for the adsorption of investigated MMI inhibitor molecule on the AMOF surface (**a**) initial MMI and AMOF, (**b**) final MMI and AMOF.

To gain a deeper understanding of the interaction between the MMI and the AMOF, MD simulation was performed to explore the corresponding adsorption mechanism. As a consequence, Figure 12 displays the view of the equilibrium configurations for the adsorption of the MMI inhibitor on the AMOF surface. The MMI was closer to the AMOF surface than the initial configuration. Additionally, the interaction energy between the MMI and AMOF was calculated. The obtained interaction energy value was −20.27 kJ/mol. Thus, the MMI can adsorb onto the AMOF surface, agreeing well with the electrochemical results. 

**Figure 13:**
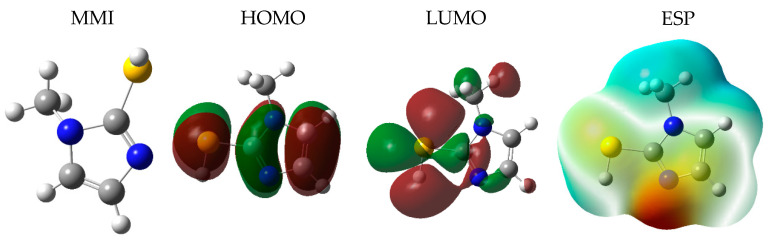
The optimized structure, frontier orbital density distributions, and electrostatic potential (ESP) map of MMI.

The electronic gap and the ionization potential (equal to |E_HOMO_|) can be associated with the corrosion inhibition properties of molecules. The HOMO energy characterizes the susceptibility of the molecule to electrophilic attack, whereas the LUMO energy is related to the electron affinity and the ability of the molecule to interact with nucleophiles. A smaller gap energy indicates a greater reactivity of the molecule and therefore a better protection against corrosion [58]. As seen in Figure 13, the HOMO was mainly located on C=C, N–C=N, and its neighboring sulfhydryl group in the MMI molecule. Furthermore, the LUMO in the MMI was constituted by S atoms of sulfhydryl groups and their neighboring carbons far from the methyl group that showed a considerable charge distribution change in this part of the inhibitor molecule. The calculated E_HOMO_ (–5.60 eV) was lower than the Fermi level of Cu (−4.96 eV), which explained the electron transition from the inhibitor to the surface. Moreover, in the electrostatic potential (ESP) map, the brownish yellow (negative) regions associated with nucleophilic reactivity and the blue (positive) regions with electrophilic reactivity were mainly distributed in the S and N atoms in the imidazole ring, respectively. Therefore, assuming that the MMI contains three major adsorption sites, including two N atoms and the S atom, is reasonable. Furthermore, to express the chemical reactivity of the MMI, the global electrophilicity index (ω), electronegativity (χ), dipole moment (μ), and hardness (η) of the MMI molecule were calculated as 1.16 eV, −2.63 eV, 3.01 D, and 2.97 eV, respectively. E_LUMO_ (0.34 eV) and E_HOMO_ (–5.60 eV) were used to calculate the gap energy, which has a value of 5.94 eV. These results show that N–N bonds and the SH group play important roles in the molecular activity of the MMI. The computation data support the experimental results well and demonstrate the effective interaction of the MMI with Cu, leading to its good inhibition performance.

**Figure 14:**
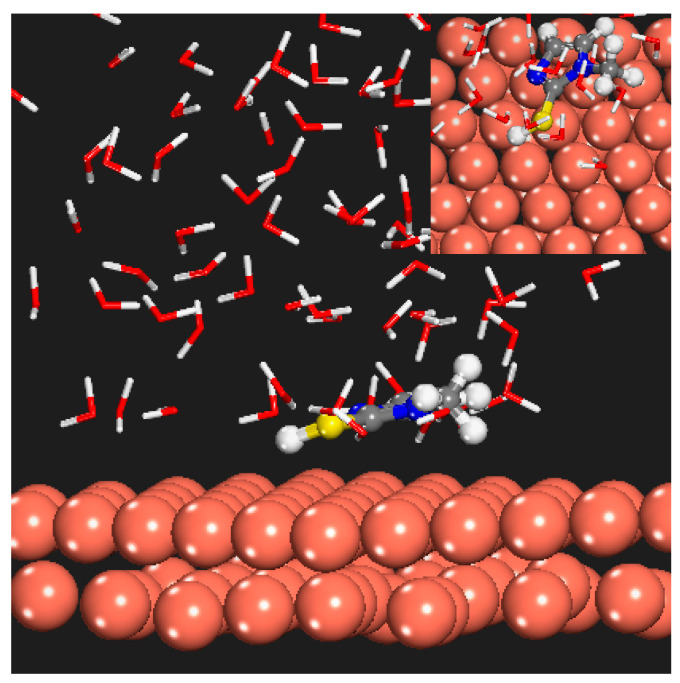
Equilibrium configurations for the adsorption of investigated MMI molecule on the Cu (111) surface (inset: on-top view).

The adsorption mechanism of the MMI on the Cu surface was further predicted by classic MD simulation. According to the equilibrium configuration of the MMI molecule adsorbed on the Cu surface, results showed that the inhibitor in the initial configuration of the simulation system was so far from the Cu surface. Subsequently, the positions of the inhibitor molecule gradually moved toward the Cu surface (Figure 14). Accordingly, the Cu surface was gradually covered by the adsorbed MMI molecules, and the corrosion process was inhibited [59]. 

## 4. Discussions

### 4.1. Interaction Mechanism of MMI and AMOF

In the medium of this study, the corrosion of copper can be expressed by the following reactions:

Cathodic: O_2_ + 2H_2_O + 4e^−^ →4OH^−^ (5)

Anodic: Cu + Cl^−^ → CuCl +e^−^ (6)

CuCl +Cl^−^ →CuCl_2_^−^ (7)

When MMI is present in the corrosive medium, it may cover the copper surface, thereby isolating the corrosive medium and inhibiting corrosion.

The SEM, FTIR, and XRD results are used to provide direct evidence to prove the interaction between the MMI inhibitor and the AMOF. In terms of theoretical calculation, the negative value of E_interaction_ further proves the MMI adsorption on the AMOF surface. The loading capacity determined by the direct dosage of the MMI onto the AMOF by TGA is 18.15%. UV-vis spectroscopy analysis is adopted to assess its release capacity. Figure 4b displays the kinetic profiles of the inhibitor release to 0.5 M of NaCl solution under a stirring environment. A progressive and sustained release with no burst effect, consisting of two distinct stages with a successively declining release rate, was observed. The delivery process of the surface-loaded inhibitor is predominantly governed by the host–guest interactions because the solution is under constant stirring during the release experiment. In this case, the most likely interaction for the MMI loaded onto the AMOF involves two situations. First, for inhibitor molecules rooted close to the framework, the forces are dominated by coordination bonding between the functionalized heterocycles and the unsaturated Zn–N sites. Second, for those far away from the framework, the forces are generated by intermolecular bonding, such as hydrogen and electrostatic bonding. 

### 4.2. Corrosion Inhibition Mechanism of Released MMI

**Figure 15:**
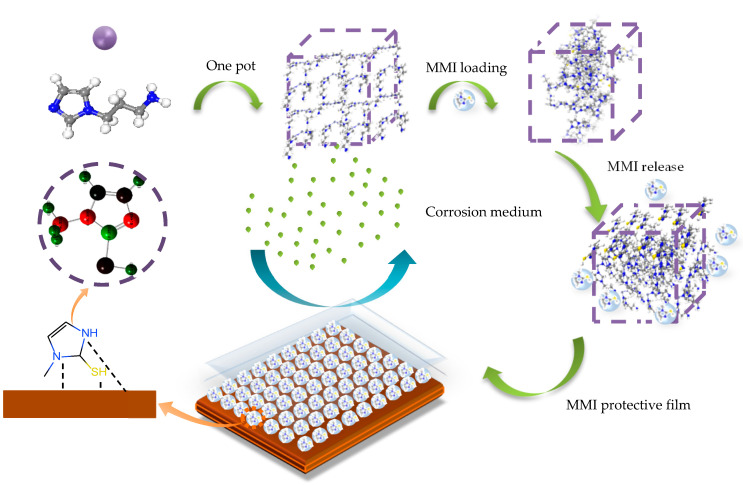
The corrosion inhibition mechanism diagram of the AMOF@MMI system for copper in 0.5 M NaCl solution.

The charge distribution data in MMI molecules from a quantum chemical calculation show that S atoms out of the ring are negative (Figure 15). N atoms on the ring have high activity because of the resonance of the molecular structure. These places are both active adsorption sites. The MMI molecule can be absorbed on the Cu surface through the imidazole ring and sulfhydryl group [53,60]. In an XPS test (Figure 10), the peaks of Cu–S bonds at 162.63 eV indicate the adsorption of the MMI on the Cu surface. In addition, the analysis of C and N can disclose the existence of C=N and C–S [61,62,63]. The peak at 402.67 eV represented a Cu–N bond, which indicates the coordination between metal and nitrogen contained in the imidazole ring [64]. Therefore, S atoms out of the imidazole ring and N atoms in the imidazole ring act as the active adsorption sites. MMI molecules are adsorbed on the Cu surface and separate the corrosion medium from the Cu surface [55,57,65]. 

## 5. Conclusions

A novel inhibition system was designed and prepared based on an AMOF and an MMI. The AMOF has a high load capacity on the MMI, and there is a strong binding force between them. The MMI could be released from the inhibition system and significantly inhibit copper corrosion.

## Figures and Tables

**Table 1:** Polarization parameters of copper in 0.5 M NaCl with AMOF@MMI at different time.

Time (h)	E_corr_ (mV vs. SCE)	b_a_ (mV/dec)	b_c_ (mV/dec)	i_corr_ (A/cm^2^)	η (%)
0	−218 ± 2	53.5 ± 1.4	−112.0 ± 6.1	(3.22 ± 0.14) × 10^−5^	/
1	−209 ± 1	60.6 ± 3.3	−119.2 ± 10.5	(1.45 ± 0.26) × 10^−5^	55.2 ± 10.1
2	−192 ± 3	55.0 ± 2.8	−125.0 ± 29.0	(7.32 ± 0.83) × 10^−6^	77.3 ± 2.6
3	−181 ± 2	52.1 ± 3.6	−127.8 ± 13.7	(6.31 ± 0.74) × 10^−6^	80.4 ± 3.2
4	−176 ± 4	53.0 ± 4.3	−126.8 ± 19.0	(4.62 ± 0.53) × 10^−6^	85.7 ± 2.2
5	−163 ± 2	61.7 ± 7.3	−129.9 ± 19.5	(3.93 ± 0.81) × 10^−6^	87.8 ± 1.8
6	−144 ± 3	55.2 ± 3.1	−179.0 ± 45.7	(3.80 ± 0.16) × 10^−6^	88.2 ± 1.4

**Table 2:** Parameters for EIS measurements with AMOF@MMI at different times.

Time (h)	Rs (Ω·cm^2^)	CPE_dl_-T (S^n1^·Ω^−1^·cm^−2^)	n_1_	*R*_ct_ (Ω·cm^2^)	CPE_f_-T (S^n2^·Ω^−1^·cm^−2^)	n_2_	*R*_f_ (Ω·cm^2^)
blank	4.3 ± 0.4	(2.26 ± 0.22) × 10^−5^	0.88 ± 0.03	1278.8 ± 16.1	(1.42 ± 0.13) × 10^−4^	0.65 ± 0.07	2276 ± 48.7
1	9.4 ± 0.6	(1.53 ± 0.79) × 10^−5^	0.82 ± 0.04	2612.1 ± 34.0	(8.19 ± 0.04) × 10^−6^	0.90 ± 0.04	4206 ± 40.2
2	10.0 ± 1.2	(1.45 ± 0.25) × 10^−5^	0.83 ± 0.06	4194.5 ± 37.1	(5.55 ± 0.05) × 10^−6^	0.96 ± 0.01	8262 ± 89.0
3	17.6 ± 0.2	(1.26 ± 0.43) × 10^−5^	0.83 ± 0.06	5474.7 ± 56.1	(4.41 ± 0.03) × 10^−6^	0.99 ± 0.05	9384 ± 89.0
4	11.3 ± 1.5	(1.10 ± 0.35) × 10^−5^	0.83 ± 0.06	7408.3 ± 72.1	(2.82 ± 0.10) × 10^−6^	0.94 ± 0.04	11,308 ± 89.0
5	12.0 ± 1.0	(1.14 ± 0.36) × 10^−5^	0.77 ± 0.06	9378.5 ± 191.8	(2.83 ± 0.04) × 10^−6^	0.97 ± 0.08	12,014 ± 301.4
6	8.1 ± 1.5	(1.09 ± 0.29) × 10^−5^	0.81 ± 0.08	11,330.8 ± 305.4	(2.55 ± 0.06) × 10^−6^	0.99 ± 0.02	13,633 ± 246.8

**Table 3:** Chemical composition of the copper surface after immersion in blank solution for 3 h and in AMOF@MMI-containing solution for 3 h and 6 h.

Sample	C 1s	O 1s	N 1s	S 2p	Cl 2p	Cu 2p	Zn 2p
blank	37.59	40.72	1	0.32	4.36	15.36	0.65
3 h	44.19	24.39	10.24	5.21	2	12.83	1.15
6 h	44.74	12.85	16.77	8.07	2.54	11.27	3.77

## Data Availability

Data is unavailable due to privacy restrictions.
